# Supplementary material for: Caring for Homebound Veterans during COVID-19 in the U.S. Department of Veterans Affairs Medical Foster Home Program
Source: Geriatrics (Basel). 2022 Jun 15;7(3):66. doi: 10.3390/geriatrics7030066 (PMC9223204; doi:10.3390/geriatrics7030066)
Supplement: Supplementary file 1 [file geriatrics-07-00066-s001.zip › geriatrics-1724477-supplementary.pdf]

2021 MFH Evaluation Interview Guide <u>HBPC Providers</u>

Interviewer:	Participant Number:	Date:
Time Start:	Time End:	Duration:

Hello [Interview participant name],

My name is (interviewer name) and I am part of the VA evaluation team in Denver, Colorado. Our evaluation provides ongoing updates to the Office of Rural Health (ORH) on the progress of Medical Foster Home (MFH) programs that received funding to expand into more rural geographical settings. We are interested in learning about how you provided care to Veterans in MFH programs and how it may have changed over the different phases of the COVID-19 pandemic.

This interview will take about half an hour to an hour of your time. What we learn from these interviews will be used to inform and further expand the MFH program in rural communities. Remember, your responses are confidential, you will not be identified in any reports, presentations, or publications. Your participation is voluntary. You can stop the interview at any time and please let me know if there's a question that you would prefer to not answer.

I would like to record the interview. The recording will be stored directly to an access file on the VA intranet. Is this okay with you? Okay, to confirm, I'm starting the recording. Is this ok with you?

If Yes, Hit Record.

Okay, to confirm, I'm starting the recording. Is this ok with you?

Grounded prompts:

1. ***What do you mean by ____________?***
2. ***Tell me more about ____________ ?***
3. ***Can you give me an example of ____________?***
4. ***Can you tell me about a time when ____________?***
5. ***Who____________?***
6. ***When____________?***
7. ***Where____________?***

1. To begin, we have a few questions about your role on your HBPC team.
   a. What is your HBPC Specialty?
   b. How long have you worked with HBPC?
   c. How long have you worked with Medical Foster Homes?

2. Please tell me about your experience providing care to Veterans in MFH programs throughout the pandemic.
   a. Did any of the MFH Veterans or Caregivers test positive for COVID 19
      i. If yes, can you explain the outcome of the positive diagnosis and how HBPC provided care to that home.

3. Tell me about any differences in how care was delivered to Veterans in MFHs and Veterans in their own residences (your HBPC Veterans).

4. Prior to COVID, to what extent was telehealth (video, VVC, phone, other) being used in MFHs by your HBPC team? How about with HBPC Veterans?
    a. How did telehealth use in MFHs change over time during the pandemic?
    b. What did you find challenging in providing care to Veterans in MFHs using telehealth?
        i. How did you respond to these challenges?
    c. What has facilitated telehealth use with MFH Caregivers?
    d. What has facilitated telehealth use with MFH Veterans?

5. What, if any, training did HBPC provide to MFH Caregivers and Veterans on using telehealth technologies?

6. Under what circumstances during the pandemic was providing in-person visits necessary for MFHs? Please provide an example.
    a. If you stopped doing in person visits initially during the pandemic, when did you start them again?

7. In your opinion, what were caregivers the most concerned about throughout the pandemic?
    a. How did you address these concerns?

8. While working with Veterans during the pandemic, what were you the most worried about, in relation to COVID?
    a. Were there Veterans you were most worried about?
    b. Is there anything that would have made you more comfortable as health care provider during the pandemic?
    c. Did you have personal concerns?
    d. Tell me about your own coping strategies during the pandemic.

9. What if any HBPC activities in MFHs were stopped?
    a. Which HBPC activities in MFHs continued?

10. Do you know if new Veterans have been admitted into a MFH during the pandemic? Can you tell me about what this process looked like?
    a. Where did the Veteran come from? Nursing Homes? Own residence?
    b. Were there challenges, if any?
    c. What facilitated this process?

11. Over the course of the pandemic, did you experience any changes in staffing in HBPC?
    a. If yes, how did this impact your work during the pandemic?
    b. Do you think this had any impact on Veterans in MFHs? If so, how?

12. Besides what we have already discussed, what other adjustments were made in how care was delivered to Veterans by your VA facility?
    a. Were these changes effective? Why or why not?

  b. How would you describe the communication, related to the pandemic, from your local VA facility to your HBPC team?
  c. Were supplies provided timely and adequately?
  d. Were resources or supplies lacking?

13. Were there any directions or guidelines from local or state authorities that you had to follow?
14. Are there any local guidelines in place for COVID testing that you have been following?
  a. What, if anything, have you heard from the VA about the ability to get the vaccine from the VA/for the Veterans in your care?
  b. Do you intend to get the vaccine or have already gotten it? Why or why not?
  c. How would you describe COVID vaccination hesitancy in the community? Among Veterans? Among MFH caregivers?

15. How prepared do you think MFH programs are now to continue providing care during the pandemic, and handle surges in COVID cases?

16. What do you think is still needed to assist MFH programs for continuing to care for Veterans during the pandemic?
  a. How about in handling other emergencies?

17. What important lessons did you learn over the changing phases of the pandemic?

18. Is there anything else you would like to share with us about your experience during the pandemic?

Those are all the questions I have for you. Thank you for your time.

2021 MFH Evaluation Interview MFH Caregivers

Interviewer:	Participant Number:	Date:
Time Start:	Time End:	Duration:

Hello [Interview participant name],

My name is (interviewer name) and I am part of the VA evaluation team in Denver, Colorado. Our evaluation provides ongoing updates to the Office of Rural Health (ORH) on the progress of Medical Foster Home (MFH) programs that received funding to expand into more rural geographical settings. We are interested in learning about how you provided care to Veterans in MFH programs and how it may have changed over the different phases of the COVID-19 pandemic.

This interview will take about half an hour to an hour of your time. What we learn from these interviews will be used to inform and further expand the MFH program in rural communities. Remember, your responses are confidential, you will not be identified in any reports, presentations, or publications. Your participation is voluntary. You can stop the interview at any time and please let me know if there's a question that you would prefer to not answer.

I would like to record the interview. The recording will be stored directly to an access file on the VA intranet. Is this okay with you? Okay, to confirm, I'm starting the recording. Is this ok with you?

If Yes, Hit Record.

Okay, to confirm, I'm starting the recording. Is this ok with you?

Grounded prompts:

1. ***What do you mean by ____________?***
2. ***Tell me more about ____________ ?***
3. ***Can you give me an example of ____________?***
4. ***Can you tell me about a time when ____________?***
5. ***Who____________?***
6. ***When____________?***
7. ***Where____________?***

1. Please tell me about your experience caring for Veterans during the COVID-19 pandemic.
    a. Did you admit a new Veteran into your home during this period? What did this look like for you?
    b. Where did the Veteran come from? Nursing Home? Own home?
2. Tell me about how you stayed connected with your MFH coordinator.
    a. What do you think worked well?
    b. What do you think did not work well?
    c. Have you done any tele visits during COVID?
    d. Were tele visits between the VA and your home a new to your Veterans care?
    e. Which modes of telecommunication did you like best? (VVC, phone, video, face time, other)

3. What were the major differences between providing care before the pandemic and during the pandemic?
    a. What guidance's related to COVID were first announced by the MFH program?
        i. How did you feel when these guidance's were first announced?
    b. What preparations, if any, did you make for the pandemic?
    c. What changes did you implement in the operations of your MFH?
        i. How did these work out?
4. Tell me about the supports you had in place.
    a. Tell me about your personal supports (within and outside the household)
        i. Over the course of the pandemic, did anyone else help you out with your caregiving activities? If so, who was that? How did they help you out?
        ii. How have/did you stay connected with others who could no longer come into your home?
5. Tell me about the support you received from the VA during the pandemic.
    a. What was effective in the VA's assistance during the pandemic?
    b. What was ineffective?
    c. What do you think can be improved in the VA's response?
6. What supports were lacking or do you think would have been helpful to have?
7. We asked a little about tele visits earlier. Can you please tell me more about your experience with the use of telehealth with VA providers including HBPC teams?
    a. What were the biggest challenges you encountered during tele visits?
    b. What do you think worked well?
    c. What do you think did not work well?
8. Were there any local and or state guidelines that you had to follow?
9. Are you aware of any local guidelines in place for COVID testing?
    a. What, if anything, have you heard from the VA about the ability to get the vaccine from the VA/for the Veterans in your care?
    b. Do you intend to get the vaccine or have already gotten it? Why or why not?
    c. How would you describe COVID vaccination hesitancy in your community? Among Veterans? Among caregivers?
10. What changes, if any, have you made to your emergency plan?
    a. In the past, are there other disasters you have managed while being a caregiver? (I.e. hurricane, flooding, fires?)
    b. Were you managing any other unusual events during the pandemic?

11. During the pandemic was there a particular patient/Veteran that you were most concerned about? Please provide an example.

12. Before the pandemic, did you have a recreational therapist come into the home to work with the Veterans?

    a. If yes, what sort of activities were the Veterans doing?

    b. How were these activities continued or affected by the pandemic?

    c. Were there alternatives to recreational activities provided to the Veterans during the pandemic? What were these?

13. In your experience, have you paid out of pocket for items for the Veterans that were not already accounted for in the monthly rates charged to the Veterans?

    a. Can you provide examples of these items?

14. What, if any, immediate needs do you have that would help you care for your Veterans that are not being addressed by the MFH program or HBPC team?

15. At this point, it has been several months since the pandemic began. How are you feeling now in your role as a caregiver, and overall?

16. What lessons did you learn from your experience over the course of the pandemic?

17. Is there anything else you would like to share with our team about your experience as a MFH caregiver during the pandemic?

Now I would like to ask a few demographic questions about yourself.

18. Demographics

   a. What is your age?
   b. What is your gender?
   c. What do you consider your ethnicity to be?
   d. What is your current relationship status? (Married, domestic partnership, widowed, divorced, separated, single/never married).
   e. How many years have you worked with the MFH program?
   f. What were you doing before becoming a MFH caregiver?
   g. What is your highest educational level?
   h. Tell me about others who are part of your household?
   i. Do you know if your MFH is in an area considered to be rural or urban?

Thank you for your time.

2021 MFH Evaluation Interview Guide <u>MFH Coordinators</u>

Interviewer:	Participant Number:	Date:
Time Start:	Time End:	Duration:

Hello [Interview participant name],

My name is (interviewer name) and I am part of the VA evaluation team in Denver, Colorado. Our evaluation provides ongoing updates to the Office of Rural Health (ORH) on the progress of Medical Foster Home (MFH) programs that received funding to expand into more rural geographical settings . We are interested in learning about how you provided care to Veterans in MFH programs and how it may have changed over the course of the pandemic.

This interview will take about half an hour to an hour of your time. What we learn from these interviews will be used to inform and further expand the program in rural communities and to inform future disaster preparedness and response. Remember, your responses are confidential, you will not be identified in any reports, presentations, or publications. Your participation is voluntary. You can stop the interview at any time and please let me know if there's a question that you would prefer to not answer.

As before, I would like to record the interview. The recording will be stored directly to an access file on the VA intranet. Is this okay with you? Okay, to confirm, I'm starting the recording. Is this ok with you?

If Yes, Hit Record.

Okay, to confirm, I'm starting the recording. Is this ok with you?

Grounded prompts:

1. ***What do you mean by ____________?***
   2. Tell me more about ____________ ?
   3. Can you give me an example of ____________?
   4. Can you tell me about a time when ____________?
   5. Who____________?
   6. When____________?
   7. Where____________?

1. Please tell me about how the COVID-19 pandemic has affected your work as MFH coordinator.
   a. What activities were stopped due to the pandemic?
   b. What activities have now resumed for your program?
   c. Have you resumed any in-person activities in the homes? (I.e. monthly visits) If so, when did those resume?
2. Did you admit any new Veterans into a MFH during the pandemic? Can you tell me about what this process looked like?
   a. Where did the Veteran come from? Nursing Homes? Own residence?
   b. Were there challenges, if any?
   c. What facilitated this process?
3. Did you have any Veterans become infected by COVID?

If yes,

a. If so, do you know how they contracted COVID?
b. Did the Veterans recover?
c. Are they still in your MFH?
d. Did they receive care in the MFH or were they hospitalized?
e. How did the HBPC team support the Caregiver?
f. Did the Caregiver make changes to how care is provided in the MFH?
g. Were any caregivers infected?
h. Was anyone else in the household infected?
i. What if any additional precautions have now been taken to prevent transmission of COVID-19 going forward?

4. Please tell me about the use of telehealth (VVC, phone, video, other) during the pandemic to deliver care to Veterans.

    a. Has telehealth been by phone, video visit, both? Using VVC, face time, or other ways?
    b. Has it increased or decreased?
    c. How are caregivers managing using telehealth?
    d. How are Veterans managing using telehealth?
    e. How do you think care delivery via telehealth can be improved?

5. Has HBPC resumed any in-person activities in the homes?

    a. If so, what are these?
    b. When did that restart?

6. We talked about changes in delivery of care via telehealth. What other changes has your MFH program made due to the pandemic?

    a. What changes were made to your MFHs' emergency plans?
    b. Did you facilitate those changes, or did your caregivers, or did you collaborate?

7. Were there directions or guidelines from local or state authorities that you had to follow?

8. Are there any local guidelines in place for COVID testing that you have been following?

    a. What, if anything, have you heard from the VA about the ability to get the vaccine from the VA/for the Veterans in your program?
    b. Do you intend to get the vaccine or have already gotten it? Why or why not?
    c. How would you describe COVID vaccination hesitancy in your community? Among Veterans? Among MFH caregivers?

9. How has the pandemic changed how you interact with other VA programs?

    a. Has the pandemic changed how you interact with leadership?

10. At this time, do you think your MFH program is prepared for any increases in COVID-19 cases in your area?

    a. Are there any other changes that you think still need to be made?

11. Over the course of the pandemic, some MFH programs lost homes or caregivers. Did this happen in your program?
    a. If so, tell us about those homes or caregivers.
    b. What plans if any, have been made to replace some of those homes/caregivers?
    c. What is your current census?
        i. Homes? Veterans?
        ii. Do you have Veterans currently interested in the program?
        iii. Do you have potential caregivers interested in the program?
        iv. At this point, are homes/caregivers with vacancies willing to admit new Veterans?

12. What, if any, staffing changes did you encounter over the course of the pandemic?
    a. How did these affect your MFH program?
    b. Are these staffing challenges ongoing or have they been addressed? If so, how have they been addressed?
    c. Do you currently have a PSA?
        i. What percentage time do they work with your MFH program?
        ii. How long have they been with you?
    d. Does your program currently have a recreation therapist (RT)?
        i. What percentage time do they work with your MFH program?
        ii. How long has the RT worked with your MFH program?
        iii. Can you tell me how you got your RT?
            1. If you were to lose your RT, how would you go about hiring a new one?
            2. Would you hire internally or from outside the VA?
            3. Have any in-person RT services resumed to your MFHs or are they still doing these remotely?
            4. How have these RT services worked out for the Veterans and caregivers?

13. What if any other things have been impactful to your MFH program during the pandemic that we have not talked about?

To end, I just have a few more questions about your role as MFH coordinator.

a. How long have you been with the MFH program?
b. What were you doing before working for the MFH program?

Those are all the questions I have for you. Thank you for your time.
